# Antioxidant Effects of Roasted Licorice in a Zebrafish Model and Its Mechanisms

**DOI:** 10.3390/molecules27227743

**Published:** 2022-11-10

**Authors:** Qian Zhou, Shanshan Zhang, Xue Geng, Haiqiang Jiang, Yanpeng Dai, Ping Wang, Min Hua, Qi Gao, Shiyue Lang, Lijing Hou, Dianhua Shi, Meng Zhou

**Affiliations:** 1 Shandong Academy of Chinese Medicine, Jinan 250014, China; 2 Shandong Academy of Sciences, Qilu University of Technology, Jinan 250103, China; 3 Shandong Institute for Food and Drug Control, Jinan 250101, China; 4 School of Pharmacy, Shandong University of Traditional Chinese Medicine, Jinan 250355, China; 5 The Second Hospital, Cheeloo College of Medicine, Shandong University, Jinan 250033, China

**Keywords:** roasted licorice, antioxidant, NRF2, KEAP1, UHPLC-Q-Exactive Orbitrap MS, zebrafish model

## Abstract

Licorice (Gan-Cao, licorice) is a natural antioxidant and roasted licorice is the most common processing specification used in traditional Chinese medicine prescriptions. Traditional Chinese medicine theory deems that the honey-roasting process can promote the efficacy of licorice, including tonifying the spleen and augmenting “Qi” (energy). The antioxidant activity and mechanisms underlying roasted licorice have not yet been reported. In this study, we found that roasted licorice could relieve the oxidative stress injury induced by metronidazole (MTZ) and could restrain the production of excessive reactive oxygen species (ROS) induced by 2,2′-azobis (2-methylpropionamidine) dihydrochloride (AAPH) in a zebrafish model. It was further found that roasted licorice could exert its oxidative activity by upregulating the expression of key genes such as heme oxygenase 1 (*HO-1*), NAD(P)H quinone dehydrogenase 1 (*NQO1*), glutamate–cysteine ligase modifier subunit (*GCLM*), and glutamate–cysteine ligase catalytic subunit (*GCLC*) in the nuclear factor erythroid 2-related factor 2 (NRF2) signaling pathway both in vivo and in vitro. Furthermore, consistent results were obtained showing that rat serum containing roasted licorice was estimated to reduce cell apoptosis induced by H_2_O_2_. Then, the UHPLC-Q-Exactive Orbitrap MS analysis results elucidated the chemical composition of rat plasma containing roasted licorice extracts, including ten prototype chemical components and five metabolic components. Among them, six compounds were found to have binding activity with Kelch-like ECH-associated protein 1 (KEAP1), which plays a crucial role in the transcriptional activity of NRF2, using a molecular docking simulation. The results also showed that liquiritigenin had the strongest binding ability with KEAP1. Immunofluorescence further confirmed that liquiritigenin could induce the nuclear translocation of NRF2. In summary, this study provides a better understanding of the antioxidant effect and mechanisms of roasted licorice, and lays a theoretical foundation for the development of a potential antioxidant for use in clinical practice.

## 1. Introduction

Membrane lipid oxidative damage caused by free radicals is an important factor tha leads to aging, cancer, and cardiovascular diseases. Research on antioxidants based on the biomedical free radical theory can play an important role in the prevention and treatment of diseases. Licorice, derived from the dried roots and rhizomes of *Glycyrrhiza uralensis* Fisch, is one of the most commonly used traditional Chinese medicine in clinical practice. It has the effect of tonifying the spleen and augmenting “Qi” (energy), clearing heat and detoxifying, eliminating phlegm and treating coughs, relieving pain, and moderating and harmonizing the properties of other herbs. Licorice has rich pharmacological activities and it has been widely used in many fields as a natural antioxidant. In general, there are two kinds of licorice used in prescriptions, raw and roasted. The roasting process of licorice can bring some changes in the biological activities as compared with its raw counterpart [1,2], for example, the pharmacological effects of invigorating vital energy is stronger in roasted licorice. Modern pharmacological studies have shown that licorice extracts have significant antioxidant activity [3,4,5,6]; the antioxidant components include flavonoids [7], polysaccharides [8], and triterpenes [9]. It has been reported that the antiaging [10], anticancer [11,12], and antiinflammatory effects of licorice [13] are closely related to its antioxidant activity. In the clinic, roasted licorice shows good anti-inflammation [14], neuroprotection [2], anti-allergy [1], and inducible granulocyte colony-stimulating factor secretion properties [15]. It is noteworthy that oxidative stress is related to the pathological states of inflammation and neurodegenerative disease [16]. On the basis of this background, it has been suggested that roasted licorice may also show good antioxidant activity. However, few studies have reported on the antioxidant activity of roasted licorice.

In this study, we investigated the antioxidant activity of roasted licorice using an in vitro and in vivo model, and we further investigated the possible material basis of its pharmacodynamics and molecular mechanism. Our results provide reliable evidence to clarify the antioxidant effect of roasted licorice extracts and the mechanisms involved.

## 2. Results

### 2.1. Roasted Licorice Extracts Protect against Oxidative Stress Injury and Excessive ROS Generation In Vivo

**Figure 1:**
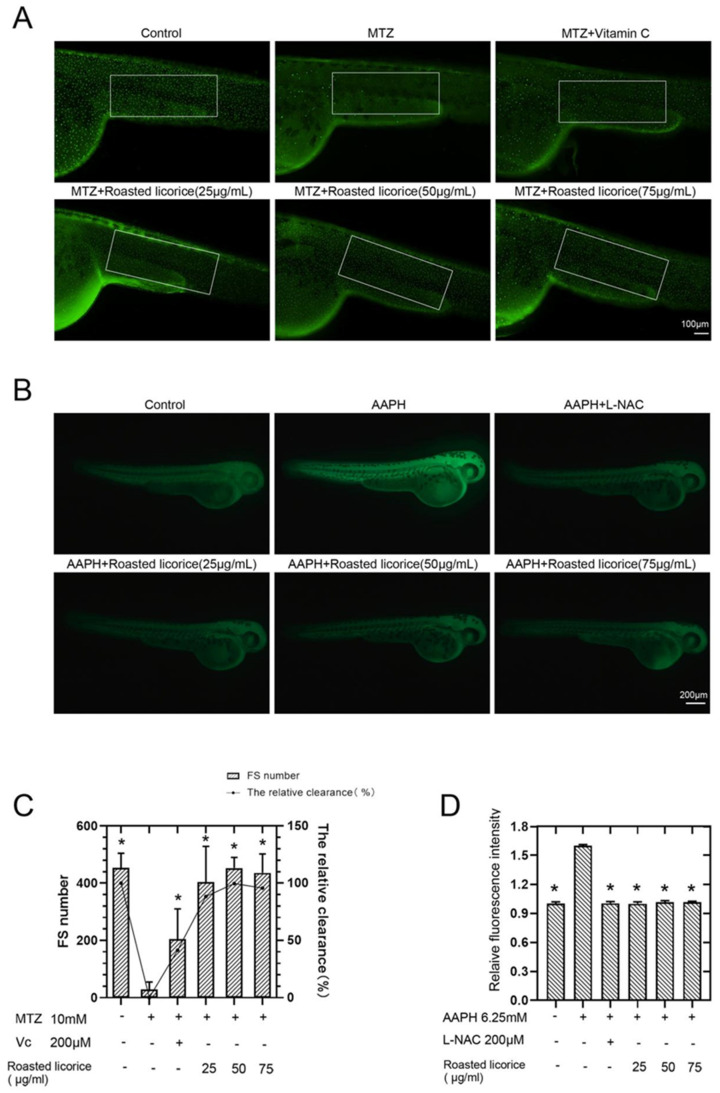
Antioxidant activities of roasted licorice extracts in zebrafish: (**A**) In vivo visualization of zebrafish skin fluorescence of drug treatment in transgenic zebrafish embryos; (**B**) in vivo visualization of ROS level of drug treatment in wild-type AB zebrafish embryos; (**C**) statistical results of fluorescence spots (FS) and the antioxidant activity of transgenic zebrafish in (**A**). The white enclosed area is the statistical range of zebrafish skin fluorescent spots; (**D**) statistical results of fluorescence intensity of wild-type AB zebrafish in (**B**). * Significant difference as compared with the MTZ or AAPH treatment group, *p* < 0.05 (*n* = 10).

According to previous research, licorice extracts have shown excellent antioxidant activity in vitro, and zebrafish has proven to be an outstanding model organism for studying ROS generation and ROS-related pathologies in oxidative stress [17]. Thus, transgenic zebrafish (ZF) embryos expressing green-fluorescent protein GFP-tagged keratin 4 protein in keratinocyte have been used as an in vivo model to study the antioxidation activity of roasted licorice extracts. Images and quantification using a GFP-tagged reporter protein are shown in Figure 1A,C, respectively. The results showed that zebrafish treated with MTZ exhibited a decreased number of GFP-positive cells in the epidermis, while roasted licorice extract treatment could revert this reduction (Figure 1A,C). Furthermore, the high, middle, and low concentration groups of roasted licorice extracts showed similar antioxidant activity (more GFP-positive cells) as compared with the MTZ treatment group (Figure 1A,C). Most importantly, the roasted licorice extract treatment was estimated to have greater antioxidant activities as compared with vitamin C, which is a proven antioxidant (Figure 1A,C).

To further estimate the capacity of roasted licorice extracts toward the inhibition of ROS accumulation, another oxidative stress model induced by AAPH [18] and an oxidation-sensitive fluorescent probe DCFH-DA were used in the study. Images and quantification using a redox-sensitive fluorescent probe are shown in Figure 1B,D, respectively. The results showed that the ROS level increased 1.6 times upon treatment with AAPH in wild-type AB zebrafish as compared with a control group (Figure 1B,D). A drug control group using N-acetyl-L-cysteine (L-NAC) was also set in the assay. L-NAC and roasted licorice extracts in different concentrations could significantly reduce the levels of ROS production (Figure 1B,D) as compared with the AAPH group.

Collectively, the abovementioned results demonstrate that the roasted licorice extracts had excellent antioxidant properties in vivo.

### 2.2. The Antioxidant Activity of Roasted Licorice Extracts Is Associated with the Transcriptional Level of Oxidant Stress-Related Genes In Vivo

**Figure 2:**
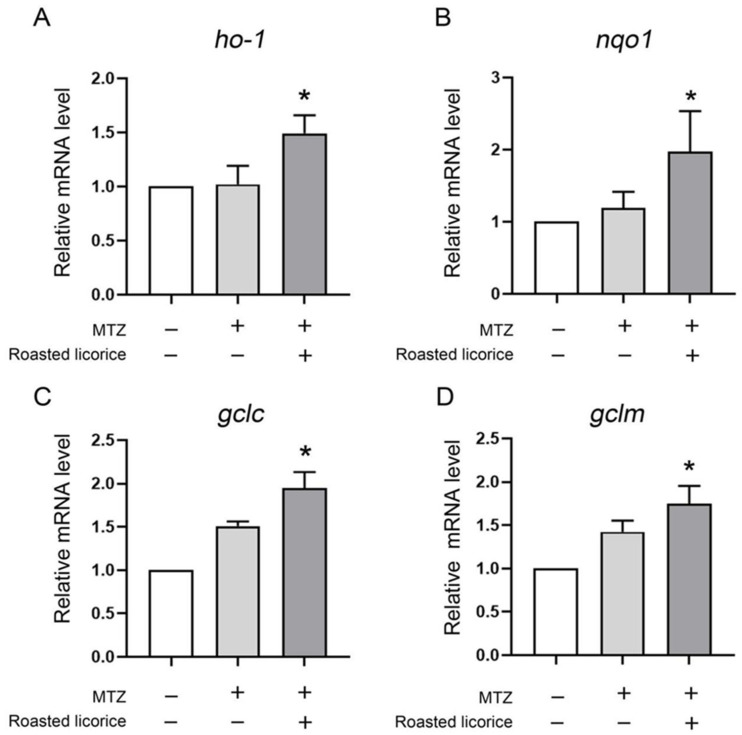
Transcriptional alteration of antioxidant genes in transgenic zebrafish Tg (Krt4-NTR:GFP) cy17. * Significant difference as compared with the MTZ treatment group, *p* < 0.05.

To further reveal the antioxidant effect in the transgenic zebrafish model, the *ho-1*, *nqo1*, *gclm*, and *gclc* genes, which play contributory roles in protecting against damage from oxidative stress, were explored. The quantitative RT-PCR (qRT-PCR) results (Figure 2) showed that roasted licorice extracts could upregulate the mRNA levels of *ho-1*, *noq1*, *gclc*, and *gclm* within the experimental concentration range (50 μg/mL) as compared with the model group. The results indicated that roasted licorice extracts could affect the transcriptional levels of antioxidative genes to ameliorate oxidant stress injury in the MTZ-induced zebrafish oxidative stress model.

### 2.3. The Antioxidant Activity of Roasted Licorice Extracts Is Associated with the Transcriptional Level of Oxidant Stress-Related Genes In Vitro

**Figure 3:**
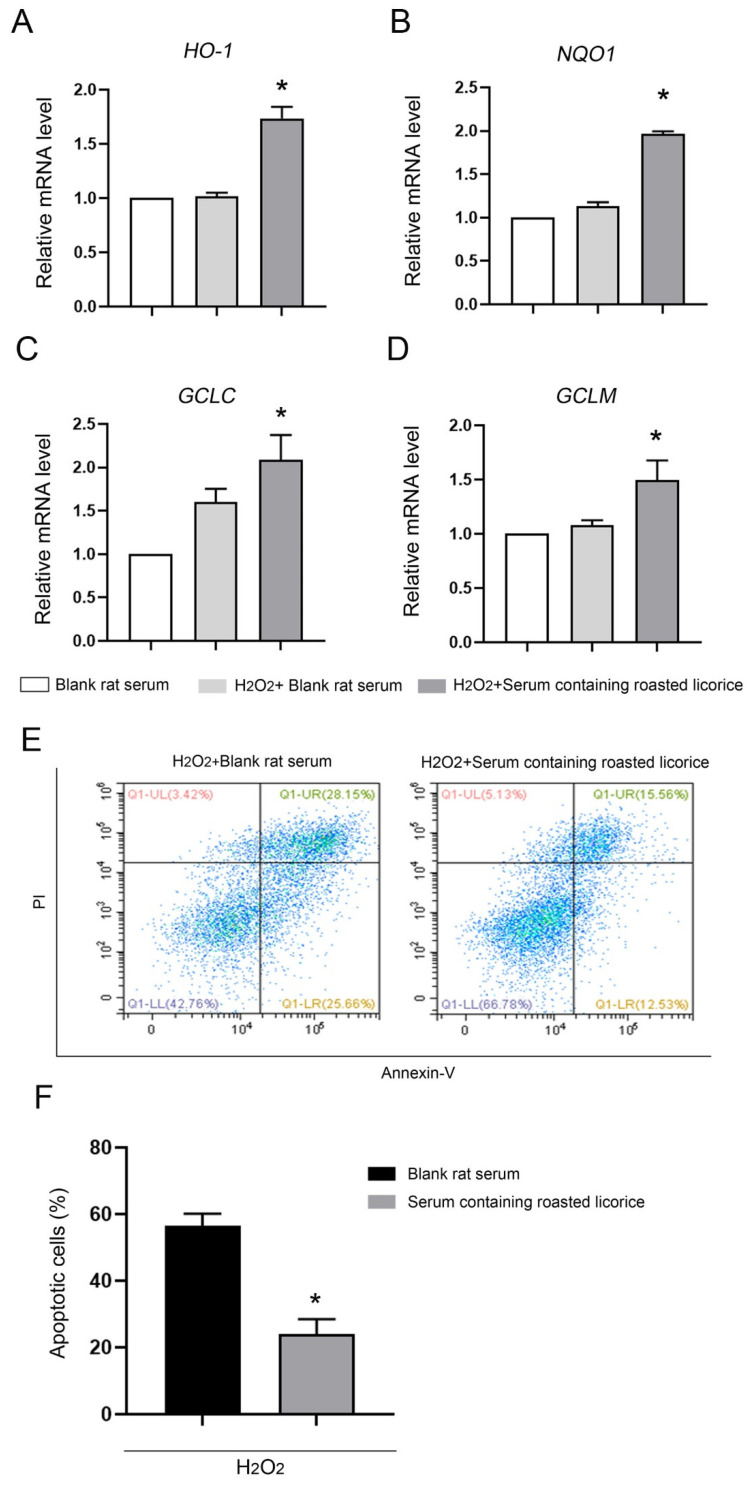
The antioxidant activity of roasted licorice extracts in vitro Transcriptional alteration of gene in Caco-2 cells: (**E**) Representative flowcharts showing that cell apoptosis was determined by flow cytometric analysis in Caco-2 cells with different treatments. Cells were stained with fluorescein isothiocyanate (FITC)-conjugated Annexin V and propidium iodide (PI); (**F**) quantification of the percentage of apoptotic cells. * Significant difference as compared with the H_2_O_2_ combined with blank rat serum treatment group, *p* < 0.05. Data were obtained from three independent experiments.

To confirm the function of roasted licorice extracts, in vitro experiments were subsequently performed using the human colon cancer cell line Caco-2, which is a common model cell line to study the oxidant stress reponse. The qRT-PCR results (Figure 3) showed that 10% serum containing roasted licorice extracts could significantly upregulate the mRNA levels of *HO-1*, *NQO1*, *GCLC*, and *GCLM* in Caco-2 cells subjected to H_2_O_2_-induced oxidative damage after treatment for 6 h. Furthermore, detection by flow cytometry (FCM) validated that treatment with serum containing roasted licorice extracts could decrease the apoptosis of Caco-2 cells after H_2_O_2_ stimulation for 24 h, which was caused by oxidant stress. These in vitro findings are consistent with the in vivo results and suggest that roasted licorice extracts could restrain the oxidative stress injury in cells.

### 2.4. Phytochemical Analysis of Roasted Licorice Extracts

**Figure 4:**
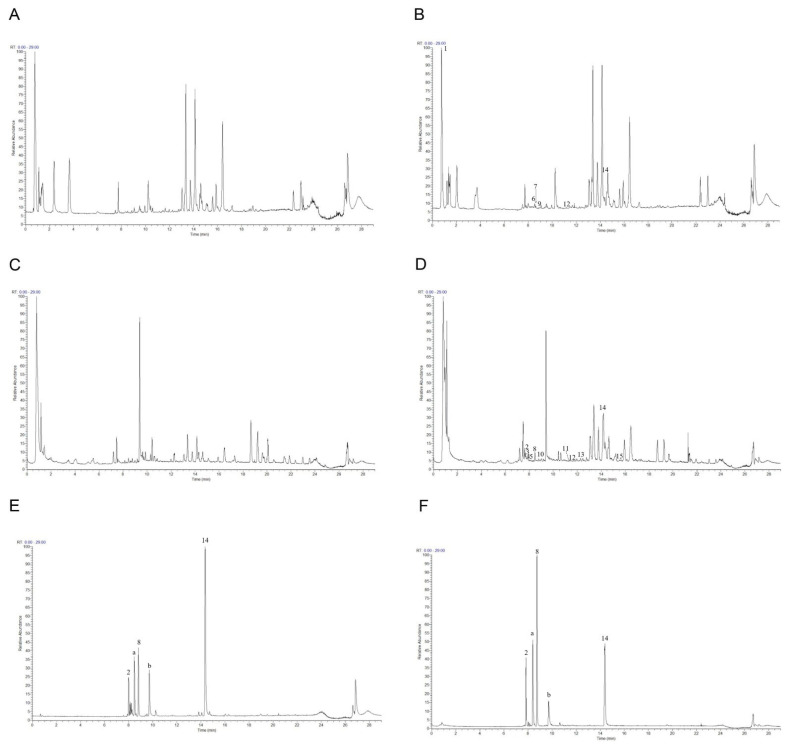
The total ion chromatograms: (**A**) Blank plasma in positive ion mode; (**B**) plasma containing roasted licorice in positive ion mode; (**C**) blank plasma in negative mode; (**D**) plasma containing roasted licorice in negative mode; (**E**) standard sample in positive ion mode; (**F**) standard sample in negative ion mode. 2, liquiritin apioside; 8, liquiritigenin; 14, glycyrrhetinic acid; a, isoliquiritin; b, glycyrrhizic acid.

To further study the underlying antioxidant mechanisms, a phytochemical analysis of roasted licorice extracts was first performed. The total ion chromatograms (TIC) are shown in Figure 4. After deducting the blank, a total of 15 transitional components of roasted licorice in plasma were identified by analyzing the retention time and mass spectrometry information, combined with literature reports and standard spectra (Table 1), including ten prototype chemical components and five metabolic components, mainly flavonoids and triterpenoids.

**Figure 5:**
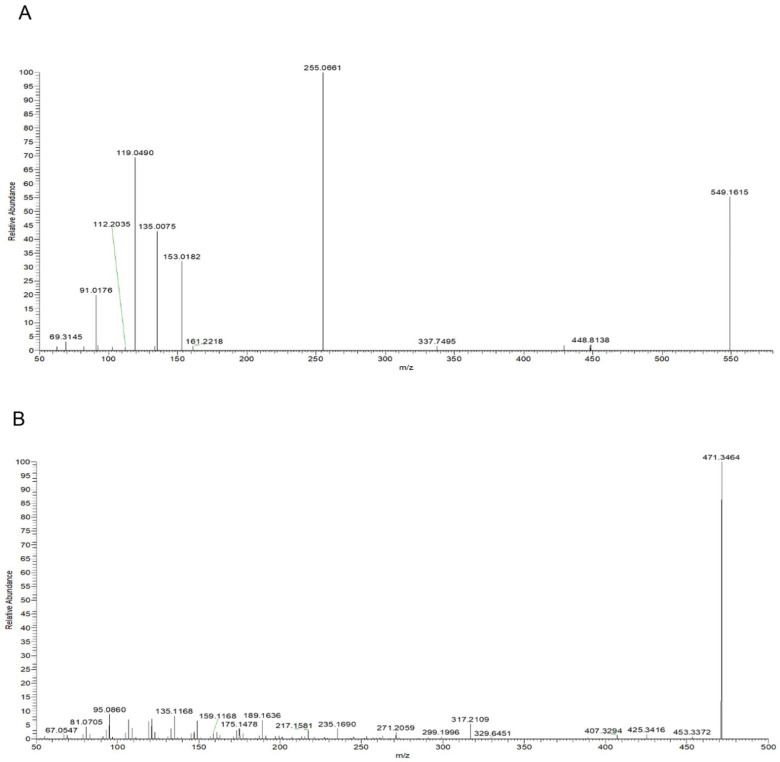
Mass spectra: (**A**) Liquiritin apioside in negative ion mode; (**B**) glycyrrhetinic Acid in positive ion mode.

Taking compound 2 as an example, according to the quasi-molecular ion peak *m*/*z* 549.1615 [M−H]^−^ generated in electrospray ionization (ESI) negative mode, the molecular formula was preliminarily inferred to be C_26_H_30_O_13_. This can also be observed in the fragmentation law of mass spectrometry that fragment ions such as 255.0661 [M−H−apiose−glucose]^−^; according to the fragmentation law, it can be judged that there were two glycosyl substitutions in the structure. In addition, in the positive ion mode, there were fragment ions such as 135.0075 and 119.0490 generated by the RAD cracking of the aglycone part, which is in line with the cracking law of flavonoids. Lastly, on the basis of the literature reports [19,20] and a comparison of the reference substance information (Figure 5A), the peak 2 compound was identified as liquiritin apioside.

Taking compound 14 as an example, we comprehensively analyzed the quasi-molecular ion peak *m*/*z* 469.3321 [M−H]^−^ in ESI negative mode and the quasi-molecular ion peak *m*/*z* 471.3464 [M+H]^+^ in ESI positive mode. The molecular formula of compound 14 was deduced to be C_30_H_46_O_4_. In addition, the compound produced fragment ions such as 189.1636, 235.1690, and 317.2109 in positive ion mode, which was in line with the fragmentation rule of glycyrrhetinic acid. Lastly, by comparing the mass spectrometry information with the reference substance glycyrrhetinic acid (Figure 5B), it was confirmed that compound 14 was glycyrrhetinic acid.

For flavonoids, compounds 3 and 6 were taken as examples; quasi-molecular ion peaks *m*/*z* 431.0999 and *m*/*z* 433.1101 were detected in positive ion mode. Additionally, fragment ions such as 119, 135/137, and 255/257, which were the same as liquiritigenin or isoliquiritigenin, were detected in the MS/MS secondary mass spectrum. The molecular weight was 176 higher than that of liquiritigenin and isoliquiritigenin, which is a typical glucuronidation combination product. Lastly, according to the comparison of their retention time and polarity, it was speculated that compound 3 was a glucuronides of liquirtigenin, and compound 6 was a glucuronides of isoliquirtigenin.

For triterpenoids, compound 12 was taken as an example; the quasi-molecular ion peak *m*/*z* 647.3789 was detected in positive ion mode and the quasi-molecular ion peak *m*/*z* 645.3643 was detected in negative ion mode. Moreover, fragments of 471.3461, 407.3277, 317.2097, and 235.1688 were detected in positive ion mode, and fragments of 469.3322 and 425.3436 were detected in negative ion mode. In the same fragment as glycyrrhetinic acid, only the molecular weight of the quasi-molecular ion peak was 176 higher than that of glycyrrhetinic acid, which is a typical glucuronidation combination metabolite [21], but its binding site could not be confirmed; thus, compound 12 could only be speculated to be the glucuronidation combination binding product of glycyrrhetinic acid.

### 2.5. The Major Phytochemicals in Roasted Licorice Extracts Show Potential Binding Activity with Keap1 according to a Molecular Docking Simulation

**Figure 6:**
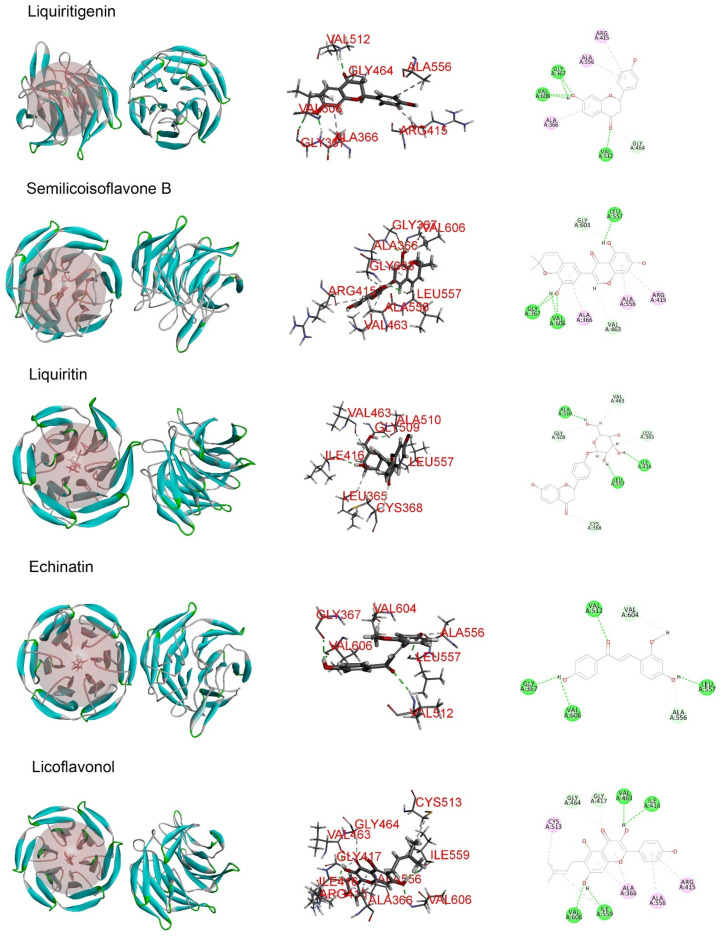

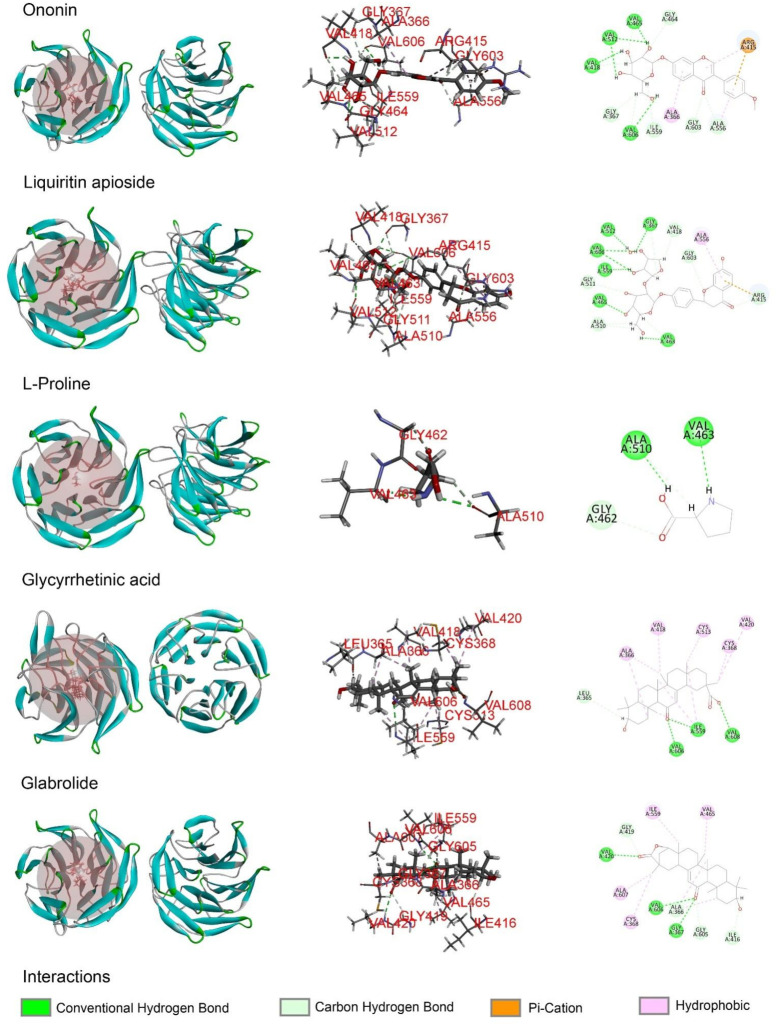
Docking simulation for the interaction between the main components of roasted licorice and KEAP1 (PDB: 4zy3), showing a general overview, local overview, and 2D overview.

Molecular docking analysis was performed in order to better understand the potential mechanisms of the antioxidant activity of the major phytochemicals in roasted licorice extracts. Since the Keap1/Nrf2/ARE signaling pathway, which has transcriptional activity of antioxidant genes such as *HO-1*, *NQO1*, *GCLM*, and *GCLC*, plays a crucial role in cell antioxidant stress [22], KEAP1 (4zy3) was chosen as a potential binding receptor in the analysis. According to the experimental results shown in Table 1, ten compounds of roasted licorice extracts were chosen as ligands. Table 2 shows the binding energies between the selected compounds and Keap1. According to the results, except for glabrolide, glycyrrhetinic acid, liquiritin apioside, and l-proline in roasted licorice extracts, other components showed stable binding ability to Keap1 with a binding energy lower than −8 kcal/mol. According to the CDocker energy, the binding ability was in the order liquiritigenin > semilicoisoflavone B > liquiritin > echinatin > licoflavonol > ononin. These results indicate that liquiritigenin showed the most stable binding to Keap1. The interaction analysis results are shown in Figure 6. The optimal binding configuration of these compounds and the receptor were obtained on the basis of the minimum binding energy after docking. According to these results, we found that liquiritigenin formed five hydrogen bond interactions with Gly367, Gly464, Val512, and Val606 and three electrostatic interactions with Ala366, Arg415, and Ala556 of Keap1. For the other compounds, semilicoisoflavone B formed seven hydrogen bonds and three electrostatic interactions with Keap1, liquiritin formed eight hydrogen bonds with Keap1, echinatin formed six hydrogen bonds with Keap1, licoflavonol formed six hydrogen bonds and six electrostatic interactions with Keap1, and ononin formed 14 hydrogen bonds and three electrostatic interactions with Keap1. The above results show that the Gly367, Gly464, Val512, and Val606 amino-acid residues in the key region of the Keap1 protein Kelch may play an important role in the antioxidant function of roasted licorice.

### 2.6. Liquiritigenin Promotes the Nuclear Translocation of NRF2

**Figure 7:**
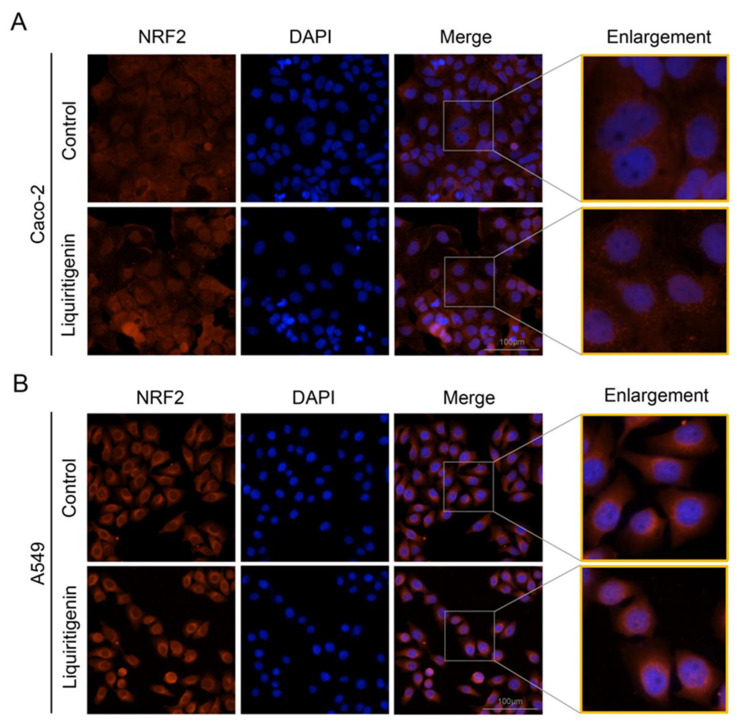
Subcellular location of NRF2 determined by immunofluorescence microscopy in Caco-2 (**A**) and A549 cells (**B**) with liquiritigenin treatment. Nuclei were revealed using 4′,6-diamidino-2 -phenylindole staining.

**Figure 8:**
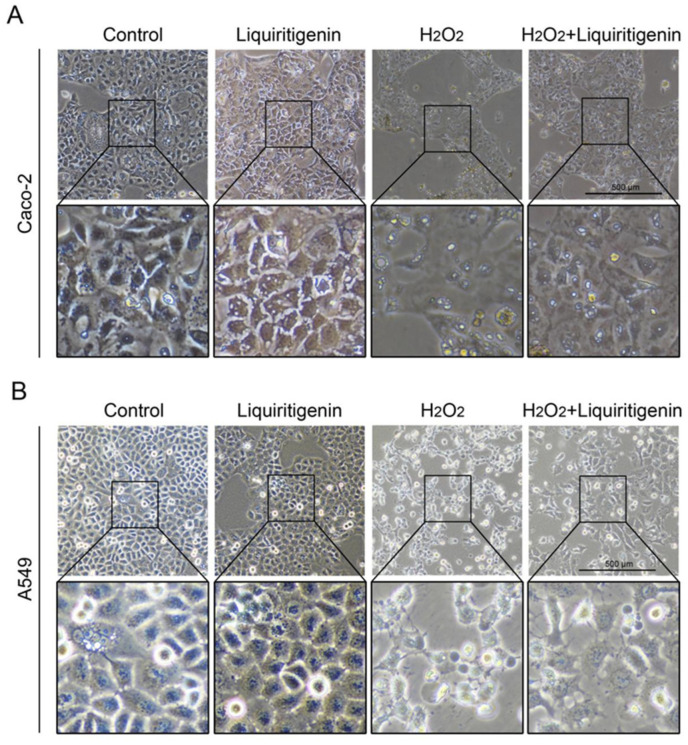
The variation of cell morphology observed under a light microscope in Caco-2 (**A**) and A549 (**B**) cells with liquiritigenin treatment against oxidative stress induced by H_2_O_2_.

In the cytoplasm, the KEAP1 protein is usually bound to NRF2 to stabilize NRF2 and promote its ubiquitination. The uncoupling KEAP1 results in the release and nucleus translocation of NRF2. According to the results of the molecular docking simulation, liquiritigenin had the strongest binding ability; thus, an immunofluorescence staining assay was performed to determine whether liquiritigenin could affect the nuclear translocation of NRF2 in cells. Human lung carcinoma cell line A549 and human colon epithelial cancer cell line Caco-2 were used in the NRF2 nuclear translocation experiment. The results showed that liquiritigenin could promote nuclear translocation in both Caco-2 (Figure 7A) and A549 cells (Figure 7B). It was further found that liquiritigenin protected the cells against H_2_O_2_-induced oxidative injury, maintaining a normal cell morphology, according to observations under a light microscope (Figure 8).

## 3. Discussion

Licorice is the traditional medicine with the highest frequency included in the Catalogue of Ancient Classic Famous Prescriptions. Among the collected 100 prescriptions, 60 prescriptions contain licorice, including 27 prescriptions of roasted licorice, six prescriptions of raw licorice, and 27 prescriptions without a clear description of the processing type. It is known that proper processing can change the curative efficacy of Chinese herbal medicine [23]; therefore, understanding the pharmacological properties of roasted licorice can contribute to the right selection of its processing method in prescriptions. Accumulating studies have suggested that licorice can clear heat and detoxify, and it is mostly used for detoxification and antibacterial effects, while roasted licorice can nourish the spleen and enrich Qi with more obvious beneficial efficacy and antioxidation effects. In this regard, the pharmacological activities of roasted licorice were studied from the point of view of antioxidation. Here, we found that roasted licorice could relieve oxidative damage of the zebrafish epidermis induced by MTZ. We further found that roasted licorice could inhibit the ROS accumulation of zebrafish induced by AAPH.

Oxidative stress, a status of excessive ROS generation exceeding the capacity of antioxidant defense, is known to be involved in a wide range of pathophysiological processes. NRF2 targets genes associated with oxidative damage in cells. During oxidative stress, NRF2 uncouples from chaperone protein KEAP1 and is rapidly transferred to the nucleus, binding with the intracellular antioxidant response elements, and initiating the expression of downstream antioxidant genes such as *HO-1*, *NQO1*, *GCLM*, and *GCLC*. In our study, we verified the upregulation of antioxidant genes at a transcriptional level induced by roasted licorice extracts in oxidative stress models in vitro and in vivo. The results indicated that roasted licorice significantly influenced the NRF2 transcriptional activity. Furthermore, serum containing roasted licorice extracts could significantly reduce the cell apoptosis in a H_2_O_2_-induced Caco-2 model. However, we cannot exclude the possible effect of roasted licorice on a non-NRF2 pathway on the basis of the current results, and the effect of roasted licorice on the post-transcriptional expression levels of these genes is unclear.

Next, the compound composition in the medicated plasma of roasted licorice was studied. Many compounds of licorice have been reported in the literature to have antioxidant activity, such as isoliquiritigenin, licochalcone A, and glycycourmarin [24,25]. In our study, the other 15 compounds in total were identified from the roasted licorice aqueous extracts in rat plasma, including ten prototype components and five metabolic components. We speculated the possible active substances in the NRF2 signaling pathway in vitro according to the molecular docking results. An important finding in this study was that liquiritigenin and five other compounds showed good interaction with KEAP1. Among the six components, liquiritigenin had the strongest binding ability.

Our findings further showed that liquiritigenin could promote the transportation of NRF2 to the nucleus and reduce cell damage induced by H_2_O_2_ in both A549 and Caco-2 cells. However, it was impossible for us to verify whether liquiritigenin specifically bound to KEAP1 and subsequently activated NRF2 and its antioxidant effect. Further studies may address this issue by constructing expression plasmids with or without mutating the predicted KEAP1 residues predicted to form favorable interactions with liquiritigenin to examine the residues critical for binding. Although further studies are needed, our study, to a certain extent, confirmed the antioxidant role of roasted licorice and identified liquiritigenin as one of its active ingredients in oxidative stress through KEAP1–NRF2 signaling.

## 4. Materials and Methods

### 4.1. Reagents

Licorice was purchased from Zhonglu Hospital in Jinan. Roasted licorice was prepared in the lab of the Shandong Academy of Chinese Medicine. A voucher specimen was deposited in the lab of the Shandong Academy of Chinese Medicine. Vitamin C was purchased from Shanghai Aladdin Bio-Chem Technology (Shanghai, China). MTZ, AAPH, and DCFH-DA were purchased from Yuanye Biological Technology (Shanghai, China) and liquiritigenin was purchased from Chengdu Biopurify Technology Development (Chengdu, China). The H_2_O_2_ solution was purchased from Sigma-Aldrich (St. Louis, MO, USA).

### 4.2. Animals

Wild-type AB zebrafish and transgenic zebrafish Tg (Krt4-NTR: GFP)^cy17^ were used in this study and maintained in the lab of the Biology Institute, Qilu University of Technology (Shandong Academy of Sciences), according to the standard procedures (Westerfield, 1994). Adult zebrafish were maintained under a 14/10 h light/dark cycle photoperiod. Embryos at 24 h were examined, and then the normal embryos were collected for further experiments.

SPF male Sprague-Dawley rats (8–12 weeks, 200 ± 20 g) were purchased from Jinan Pengyue (Jinan, China), production license number: SYXK (Lu) 20190003. The animal experiments were approved by the Ethics Committee of the Shandong Academy of Chinese Medicine (SDZYY20200101001). The animals were kept in their cages for 1 week prior to experiments to allow for acclimatization to the laboratory conditions. Conventional rodent laboratory diets were used with an unlimited supply of drinking water.

### 4.3. Cells and Culture Conditions

The Caco-2 and A549 cell lines were purchased from Procell (Wuhan, China). The Caco-2 cells were cultured in Minimum Essential Medium (Procell, Wuhan, China) supplemented with 20% fetal bovine serum (Hyclone, UT, USA). The A549 cells were cultured in Ham’s F-12K medium (Procell, Wuhan, China) supplemented with 10% fetal bovine serum (Hyclone, UT, USA). All the cells were cultured in an incubator at 37 °C with 5% CO_2_.

### 4.4. Sample Preparation

Different sample preparations are explained below.

#### 4.4.1. Roasted Licorice

First, 25 g acacia honey (purchased from Beijing Tung Jen Tang Limited) mixed with an appropriate amount of boiling water was added to 100 g of licorice (decoction, made form the dried roots and rhizomes of *Glycyrrhiza uralensis* Fisch). Then, the licorice was stir-fried for 20 min at 200 °C until the surface became deep yellow, taken out, and cooled in the air.

#### 4.4.2. Roasted Licorice Extracts for Zebrafish Model Experiment

A rough powder of roasted licorice was taken, accurately weighed, and placed in a round-bottomed flask, before being extracted by refluxing using ten-fold and eight-fold volumes of water successively. The mixture was decocted for 40 min each time before filtering. The combined filtrate was condensed to a certain centration. One part of the obtained concentrate was used for oral administration as described in Section 4.4.3 (1 g/mL, crude drug/mL) and Section 4.4.4 (0.09 g/mL, crude drug/mL), whereas the other part was used for a zebrafish model experiment after freeze-drying the powder.

#### 4.4.3. Plasma Samples for UHPLC–Q-Exactive Orbitrap MS Detection

Sixteen rats were randomly divided into a solvent control (water) group and an experimental group (*n* = 8/group), dosing intragastrically with roasted licorice extracts (1 g of crude drug/mL) and water, respectively, twice a day with an interval of 6 h for two consecutive days. The dose volume for administration was 1 mL/100 g body weight. After the last dosing on Day 2, the rats fasted overnight (water was still provided). On the third day, the rats were anesthetized with 3% sodium pentobarbital intraperitoneally at 1 h after drug administration at the same dose, and blood samples were collected from the abdominal aorta using evacuated tubes containing anticoagulant additives. The blood samples were set for 30 min and centrifuged for 10 min at room temperature. Then, plasma samples (supernatant from the blood samples) were obtained and stored at −80 °C for future use. The plasma samples described above were melted at 4 °C. A 200 μL sample was put into the centrifuge tube; then, 1 mL of methanol was added, and the sample was mixed for 2 min, before centrifuging at 12,000 rpm for 10 min. The supernatant liquid was placed in a 37 °C water bath and blow-dried with N_2_. The residue was redissolved with 1 mL of methanol, vortically mixed for 1 min, and centrifuged at 12,000 rpm for 10 min, and then the supernatant was obtained.

#### 4.4.4. Serum Samples for Cell Experiment

Sixteen rats were randomly divided into a solvent control (water) group and an experimental group (*n* = 8/group), dosing intragastrically with roasted licorice extracts (0.09 g crude drug/mL) and water, respectively, twice a day with an interval of 6 h for two consecutive days. The dose volume for administration was 1 mL/100 g body weight. After the last dosing on Day 2, the rats fasted overnight (water was still provided). On the third day, rats were anesthetized with 3% sodium pentobarbital intraperitoneally at 1 h after drug administration at the same dose, and blood samples were collected from the abdominal aorta using evacuated tubes containing anticoagulant additives. The blood samples were set for 30 min and centrifuged for 10 min at room temperature. Then, serum samples (supernatant from the blood samples) were obtained and stored at −80 °C for future use.

For cell experiments, the serum samples described above were melted at 4 °C, and sterilized by using 0.22 μm filters to remove bacteria before use.

### 4.5. Establishment of MTZ-Induced Oxidative Stress Model In Vivo

The zebrafish (Danio rerio) embryo model was chosen for model building as previously described (Kulkarni et al., 2018). Thus, the collected embryos at 24 hpf were treated using pronase (1 mg/mL) for 3 min to remove the outer layer egg membrane. Then, all embryos were randomly distributed into six-well cell culture plates (30 eggs/well with 5 mL of solution). The embryos were exposed to MTZ (10 mM), and the embryos treated with fish water instead of MTZ were used as a control. The antioxidant activities of roasted licorice were detected by co-treatmennt with MTZ and three different concentrations of extracts. The concentrations of samples were selected on the basis of our preliminary studies according to the reported evaluation standard (Zhang et al., 2019). The LC_1_ of roasted licorice extracts was 100 μg/mL; therefore, the tested concentrations of samples were chosen at 25 μg/mL, 50 μg/mL, and 75 μg/mL. The detection time was 24 h for antioxidant activity, and 6 h for qRT-PCR assay.

### 4.6. Establishment of H_2_O_2_-Induced Oxidative Stress Model In Vitro

The Caco-2 cells were seeded at an appropriate density in six-well plates, before incubating overnight. Then, the cells were treated with H_2_O_2_ (800 μM) with 10% serum containing roasted licorice or serum control (blank rat serum) for different times: 24 h for apoptosis detection assay by flow cytometry and 6 h for qRT-PCR assay.

### 4.7. Detection of Antioxidant Activities In Vivo

The in vivo antioxidant activities were determined using two methods. Firstly, the antioxidant activities of samples were evaluated using MTZ-induced ROS generation in the zebrafish model according to methods previously reported (Zhang et al., 2018). After incubation at 28 °C, the zebrafish at 48 hpf were anesthetized and observed using a BX53 Bio Imaging Navigator instrument (Olympus, Tokyo, Japan). The fluorescence spots (FS) of zebrafish were counted using Image-Pro Plus. The antioxidant activity of samples was determined using Equation (1).

(1) $$The\;relative\;antioxidant\;activity\left(\%\right)=\frac{\left(C1-C2\right)}{\left(C0-C2\right)}\times 100\%$$

where *C*0 represents the number of FS in the control group, *C*1 represents the number of FS for the group to be calculated, and *C*2 represents the number of FS in the MTZ group.

Secondly, oxidative stress-induced intracellular ROS generation was measured using another oxidative stress model according to a method previously reported (Kim et al., 2014) with some modification. The collected embryos at 24 hpf were treated with pronase (1 mg/mL) to remove the outer layer egg membrane. Then, all embryos were randomly distributed into 24-well cell culture plates (10 eggs/well with 2 mL of solution). The embryos were exposed to AAPH (6.25 mM) for 24 hpf to induce oxidative stress, and the embryos treated with fish water instead of AAPH were used as a control. The antioxidant activities of roasted licorice were detected by co-treatment with AAPH and three different concentrations of roasted licorice extracts. The concentrations of samples were the same as for the MTZ-induced zebrafish model. Then, DCFH-DA solution (20 μg/mL) was added to the plate. Next, the zebrafish were anesthetized and observed using a BX53 Bio Imaging Navigator instrument (Olympus, Tokyo, Japan) after incubation with DCFH-DA for 1 h in the dark at 37 °C. The fluorescence intensity of individual zebrafish was quantified using the Image J program.

### 4.8. Quantitative Real-Time PCR (qRT-PCR)

The total RNA of the treated zebrafish embryo and Caco-2 cells was extracted using a Total Tissue RNA Isolation kit (Vazyme, Nanjing, China), and then used to synthesize cDNA using the HiScript III RT SuperMix for qPCR (+gDNA wiper) (Vazyme, Nanjing, China), according to the manufacturer’s instructions. The qRT-PCR was performed in a CFX96 Real-Time System (BIO-RAD, Hercules, CA, USA) using the SYBR^®^ Green Pro Taq HS Premix II (AG, Changsha, China). The primers, shown in Table 3, were synthetized by the Shanghai Partheno Biotechnology Co., Ltd. (Shanghai, China), and β-actin was used as an internal control. The relative levels of gene expression were calculated using the 2^−ΔΔCt^ method (Livak and Schmittgen, 2001). All the samples were analyzed with three biological replicates.

### 4.9. Apoptosis Detection

Flow cytometry was used to detect the cell apoptosis of the Caco-2 oxidative stress model using an Annexin V–FITC/PI apoptosis detection kit (BD Biosciences, San Jose, CA, USA), according to the reference manual. The flow cytometry assay was performed using a flow cytometer (Beckman Coulter, Miami, FL, USA).

### 4.10. Identification of Compounds Using UHPLC–Q-Exactive Orbitrap MS

Compounds analysis was performed using a quadrupole-electrostatic field orbitrap high-resolution mass spectrometer coupled with liquid chromatography (Thermo Fisher Scientific Co., Ltd, Waltham, MA, USA). A Waters ACQUITY UPLC BEH C_18_ column (2.1 × 100 mm, 1.7 μm) was used for sample separation. Distilled water containing 0.05% formic acid was used as solvent A, and acetonitrile was used as solvent B.

The elution conditions were as follows: from 5% B to 10% B for 4.0 min, from 10% B to 30% B for 2.0 min, from 30% B to 50% B for 2.0 min, from 50% B to 70% B for 5.0 min, from 70% B to 75% B for 4.0 min, from 75% B to 90% B for 5.0 min, from 90% B to 100% B for 1.5 min, maintained at 100% B for 1.0 min, and from 100% B to 5% B for 1.5 min, maintained at 5% B for 3.0 min, The flow rate was 0.3 mL/min. The column temperature was set at 40 °C. The injection volume of the sample was 2 μL.

The ionization mode of positive and negative ion detection using an electrospray ion source was adopted. The atomization gas was high-purity nitrogen (N_2_), and the collision gas was high-purity helium (He). The full scan and data dependent MS^2^ mode were carried out for identification.The scanning range was *m*/*z*: 80~1200. The sheath pressure was 40 flow units, auxiliary pressure five flow units, spray voltage was 3.0 kV, ion transfer tube temperature was 300 °C, auxiliary gas heating temperature was 320 °C, and lens voltage was 50 V. The resolution was 70,000 forprimary mass spectrometry, and 17,500 for secondary mass spectrometry.

An appropriate amount of reference standard was accurately weighed, and methanol was added to prepare a mixed reference substance solution with a concentration of 0.04 mg/mL liquiritin apioside, 0.11 mg/mL liquiritigenin, 0.05 mg/mL isoliquiritin, 0.05 mg/mL glycyrrhizic acid, and 0.11 mg/mL glycyrrhetinic acid, filtered using a 0.22 μm microporous membrane.

### 4.11. Molecular Docking in Silico

KEAP1, a key protein of the NRF2 signaling pathway, was used as the receptor in molecular docking analysis. The crystal structures of docking molecules were obtained from the Protein Data Bank (https://www.rcsb.org/, access on 14 October 2021). Main compounds of roasted licorice including echinatin, glabrolide, glycyrrhetinic acid, licoflavonol, liquiritigenin, liquiritin apioside, liquiritin, l-proline, ononin, and semilicoisoflavone B, were used as ligands in the molecular docking analysis. The 3D structure of all ligands was constructed using Chem3D Pro 14.0 after energy minimization. Discovery studio 2016 (DS2016, Accelrys, San Diego, CA, USA) was used for molecular docking. The automated molecular docking was performed using the CDOCKER module. The values of -CDocker energy and -CDocker interaction energy were used as evaluation criteria.

### 4.12. Immunofluorescence

The Caco-2 and A549 cells were treated with liquiritigenin (50 μM) for 6 h, washed with phosphate-buffered saline (PBS, pH = 7.2), and fixed. Then, these cells were stained with anti-NRF2 antibody (1:100; Proteintech, Chicago, IL, USA) after blocking with 1% bovine serum albumin (Sigma-Aldrich, St. Louis, MO, USA) in PBS for 30 min at room temperature, followed by washing the cells four times with PBS. Finally, fluorescence-label secondary antibody was added, and the cells were embedded into gelatine solution. Immunofluorescence staining was performed using a Ti2-A fluorescence microscope (NIKON, Tokyo, Japan).

### 4.13. Statistical Analysis

All the data were represented as the means ± standard deviations (SDs). ANOVA was performed using SPSS 19.0 for significance analysis followed by Dunnett’s t test. The differences were considered statistically significant at *p* < 0.05.

## 5. Conclusions

**Figure 9:**
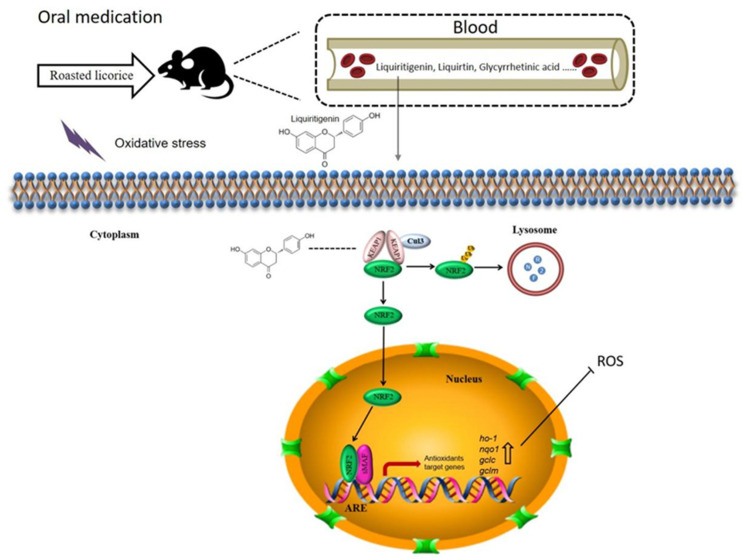
Proposed mechanisms of antioxidant activities of roasted licorice. Liquiritigenin, liquiritin, glycyrrhetinic acid, and other components could be detected in the blood after oral processing of roasted licorice in rats. In this study, we found that liquiritigenin is an important antioxidant component of the roasted licorice. In the cytoplasm, KEAP1 protein is usually bound to NRF2 to stabilize it in the cytoplasm. Liquiritigenin can bind to KEAP1, causing NRF2 to release from the protein complex and translocate into the nucleus. In the nucleus, NRF2 binds to antioxidant response elements (AREs) within the promoters of the *nqo1*, *ho-1*, *gclc*, and *gclm* genes, initiating the antioxidative response, which in turn decreases intracellular ROS production and alleviates oxidative stress-induced cytotoxicity.

In this study, we investigated the antioxidant activity of roasted licorice extracts using in vitro and in vivo models, and we predicted and verified their active ingredients. Roasted licorice extracts could alleviate oxidative stress injury by inducing inhibition of ROS generation and cell apoptosis. They were found to exert antioxidant activity by regulating the expression of antioxidant-related genes in the KEAP1/NRF2 signaling pathway. Furthermore, according to chemical, in silico, and biological methods, liquiritigenin was estimated as a major ingredient of roasted licorice extracts and a potential antioxidant by regulating the nuclear tanslocation of NRF2. In summary, our results provide reliable evidence to clarify the antioxidant effect of roasted licorice extracts and the mechanisms involved (Figure 9).

## Figures and Tables

**Table 1:** Identification results of components of roasted licorice in rat plasma.

Peak	Rt/min	Molecular Formula	Compound	Ion Mode	*m*/*z*	ppm	Secondary Fragment
1	0.85	C_5_H_9_NO_2_	l-Proline	[M+H]^+^	116.0708	1.335	116.0708, 70.0657, 68.0601
2	7.8	C_26_H_30_O_13_	Liquiritin apioside	[M−H]^−^	549.1615	2.135	549.1615, 255.0661, 153.0162, 135.0075, 119.0490
3	7.81	C_21_H_21_O_10_	Glucuronides of liquirtigenin	[M−H]^−^	431.0999	2.607	431.0999, 255.0663, 153.0184, 135.0077, 119.0491
4	7.85	C_21_H_22_O_9_	Liquiritin	[M−H]^−^	417.1191	2.568	417.1191, 255.0665, 153.0184, 135.0078, 119.0492
5	8.2	C_15_H_11_0_7_S	Sulfates of liquirtigenin	[M−H]^−^	335.0233	3.941	335.0233, 255.0661, 153.0183, 135.0076, 119.0490
6	8.56	C_21_H_21_O_10_	Glucuronides of isoliquirtigenin	[M+H]^+^	433.1101	−6.426	433.1101, 257.0803, 239.0692, 147.0438, 137.0232
7	8.63	C_22_H_22_O_9_	Ononin	[M+H]^+^	431.1331	−1.18	431.1331, 269.0804, 254.0570, 213.0905, 197.0594, 137.0233, 118.0413
8	8.64	C_15_H_12_O_4_	Liquiritigenin	[M−H]^−^	255.0654	0.842	255.0654, 153.0182, 149.0233, 135.0076, 119.0490
9	9.05	C_16_H_14_O_4_	Echinatin	[M+H]^+^	271.0956	−3.451	271.0956, 256.0713, 161.0594, 137.0596, 123.0441
10	9.06	C_15_H_11_0_7_S	Sulfates of isoliquirtigenin	[M−H]^−^	335.0232	3.553	335.0232, 255.0660, 153.0182, 135.0076, 119.0490
11	11.28	C_20_H_18_O_6_	Licoflavonol	[M−H]^−^	353.1029	2.762	353.1029, 243.1028, 201.0919, 83.0123
12	11.32	C_36_H_53_O_10_	Glucuronides of glycyrrhetinic scid	[M+H]^+^	647.3789	−0.099	647.3789, 471.3461, 407.3277, 317.2097, 235.1688
				[M−H]^−^	645.3643	1.512	645.3643, 469.3322, 425.3436
13	12.3	C_20_H_16_O_6_	Semilicoisoflavone B	[M−H]^−^	351.0873	2.750	351.0873, 307.0961, 283.0977, 265.0872, 241.0864, 199.0758, 83.0124
14	14.42	C_30_H_46_O_4_	Glycyrrhetinic acid	[M+H]^+^	471.3464	−1.096	471.3464, 453.3372, 407.3294, 317.2109, 271.2059, 235.1690, 189.1636
				[M−H]^−^	469.3321	1.883	469.3321, 425.3422, 355.2634, 161.3441
15	15.55	C_30_H_44_O_4_	Glabrolide	[M−H]^−^	467.3167	2.405	467.3167, 423.3258, 407.2991, 353.2492

**Table 2:** Docking energies for optimal conformation of main components of roasted licorice with respect to KEAP1.

	Receptors	-CDocker Energy (kcal/mol)	-CDocker Interaction Energy (kcal/mol)
Ligands			
Echinatin		25.4205	42.3573
Glabrolide		−43.6623	59.5078
Glycyrrhetinic acid		−24.6014	64.0249
Licoflavonol		25.0266	51.83
Liquiritigenin		36.1318	40.8838
Liquiritin apioside		7.21328	75.059
Liquiritin		29.3572	58.8813
l-Proline		4.2125	21.246
Ononin		23.0989	63.571
Semilicoisoflavone B		31.6962	48.8378

**Table 3:** The sequences of gene primers.

	Genes	Forward	Reverse
Zebrafish	*ho-1*	5′–AAGCAAAGCGGCAGAGAAC–3′	5′–TGGAGCAGTCAGATGAAGTGT–3′
	*nqo1*	5′–CTGGGTGGTGTGTTTGAAGAA–3′	5′–GCTGTGGTAATGCCGTAGG–3′
	*gclm*	5′–TCGCTCCTCCTTCTCTTCC–3′	5′–CCGATGGCAGCAATCTTCT–3′
	*gclc*	5′–CGGATGGAGAGTGGAGTTCA–3′	5′–TTCGCTTCTGGGCTACCTT–3′
Human	*HO* *-1*	5′–AAGACTGCGTTCCTGCTCAAC–3′	5′–AAAGCCCTACAGCAACTGTCG–3′
	*NQO* *1*	5′–GAAGAGCACTGATCGTACTGGC–3′	5′–GGATACTGAAAGTTCGCAGGG–3′
	*GCLM*	5′–CATTTACAGCCTTACTGGGAGG–3′	5′–ATGCAGTCAAATCTGGTGGCA–3′
	*GCLC*	5′–GGCACAAGGACGTTCTCAAGT–3′	5′–CAGACAGGACCAACCGGAC–3′

## Data Availability

The data that support the findings of this work are available from the corresponding author upon reasonable request.
